# Supplementary material for: The Arf-GEF Steppke promotes F-actin accumulation, cell protrusions and tissue sealing during Drosophila dorsal closure
Source: PLoS One. 2020 Nov 13;15(11):e0239357. doi: 10.1371/journal.pone.0239357 (PMC7665897; doi:10.1371/journal.pone.0239357)
Supplement: Attachment — Submitted filename: Response to Reviewers.pdf [file pone.0239357.s002.pdf]

We thank both Reviewers for their constructive feedback. We have addressed their comments with revisions/additions to the text, additional quantifications, and adjustments to a figure. The changes are described below and are also indicated with tracked changes and comment boxes in the file "Revised Manuscript with Track Changes". The line numbers below are for this file.

Responses for Reviewer #1:

1. The authors show that there is less F-actin accumulation in the LE of step mutant embryos at halfway closure. Does this happen early in formation of the actomyosin cable or later? In other words, can the authors comment on if Step is required for early F-actin enrichment at the cable, or if Step promotes F-actin accumulation during closure?

The Reviewer raises interesting questions about the initial assembly and later maintenance of the leading edge cable. Our analyses focused on stages of dorsal closure after initial cable assembly. We examined two markers of F-actin in both live and fixed samples, and observed that Step is required for normal F-actin levels in the cable at both the onset of canthus sealing and during sealing of the midline. We did not study the early assembly of the cable. We have added a sentence at line 500 to clarify this point (indicated with comment box "R1 comment 1"):

"Whether Step is required for the initial assembly of LE F-actin-based structures and/or the later augmentation of the structures will be interesting to address."

2. In Figure 2E, and results (lines 230-232), the authors state that the step mutant protrusions are abnormally short. In the images for step mutants, it is a little hard to see any protrusion at all. Instead, there seems to be a focus of Moe-accumulation. This may be due to the resolution of the movie images, but the authors could either show a different set of movie stills or explain why this Moe-accumulation is considered a protrusion.

Our minimum basis for quantifying a local leading edge F-actin accumulation as a protrusion was that its length was at least 0.5µm (as stated in the Methods and the legends for Figures 2 and 3). Characterization of such structures as a protrusion could be argued, but we prefer to include them in our counts of residual protrusions in the mutants rather than ignoring them (we may thus be underestimating the disruption to protrusions in the mutants). The images in Figure 2E are intended to show that the smallest F-actin structures we included in our counts undergo assembly and disassembly, as expected for a protrusion. Examples of larger protrusions in the mutants are shown in Figure 2C and Figure 3E. We have revised the sentence at line 294 to clarify this point (indicated with comment box "R1 comment 2"):

"The protrusions present in step mutants were often abnormally short, but even the shortest structures that we included in our protrusion counts displayed phases of growth and decay (Fig 2E)."

3. The authors state that "Step is necessary for effective localization of Ena to LE cell-cell junctions" (Fig. 4B; p. 13 lines 280-291). To me, it seems like Ena is still found at cell-cell junctions but the levels are abnormal (maybe some with lower levels and others with normal levels). Can the authors tell if Ena still localizes to all LE cell-cell junctions?

We agree with the Reviewer's interpretation of the Ena localization in *step* mutants. It seems that Ena can still localize to leading edge cell-cell junctions in the mutants, but that Ena levels at these sites are

reduced to varying degrees compared with controls. The strongest reductions correlated with abnormal cell shapes, but even in cells with relatively normal shapes the levels of Ena at LE cell-cell junctions were significantly reduced compared to similarly shaped control cells. To clarify these analyses, we have revised sentences starting at line 387 (indicated with comment box “R1 comment 3”):

“At sites of LE cell shape distortions, the enrichment of Ena to LE cell-cell junctions was much lower than controls (Fig 4B). To assess Ena localization separately from cell shape defects, we examined cells with normal rectangular shapes and although Ena localization to LE cell-cell junctions was less affected than in abnormally shaped cells it was still significantly reduced compared to similarly shaped cells of sibling controls (Fig 4B, red brackets and quantification). Thus, *step* mutants display reduced enrichment of Ena to LE cell-cell junctions in both normally and abnormally shaped LE cells, although the strongest reductions to the Ena enrichment correlated with the strongest disruptions to LE cell shape.”

4. The sufficiency of Step to induce broad lamellipodia is very interesting. Did the authors quantify how many ectopic lamellae are found in each LE, rather than just % of embryos with ectopic protrusions? Does Step overexpression induce additional filopodial-like protrusions or does Step just induce broad lamellipodial-like protrusions?

We conducted this analysis, and have added the following text at line 441 (indicated with comment box “R1 comment 4”):

“Quantification of LEs with lengths of 54.3-113.8 µm revealed that 73.6 ± 32.7% of LE length was occupied by the abnormal lamellar structures in GFP-Step overexpression embryos (N=23), whereas these abnormal structures were not observed along the length of GFP overexpression control embryos (N=24). We also quantified the numbers of F-tractin-tdTomato decorated actin bundles along the same LEs (counting those with a length of 0.5 µm or greater) and these numbers were indistinguishable between the embryos overexpressing GFP-Step (0.373 ± 0.132 bundles/µm) and GFP (0.405 ± 0.151 bundles/µm).”

Minor comments:
1. For the graphical representation of most data, the authors show each data point. This is very helpful. However, it is often useful to show the mean and SD, for example with a box-and-whisker plot. The authors may want to consider showing this, along with the individual points of data (e.g. Fig. 2B,D,F; Fig. 4B).

Thank you. We agree that it is useful to provide as much information as possible. For all data shown as scatter plots (Figs 1B, 1C, 2B, 2D, 2F, 3B, 3D, 3E, and 4B), we have now also added mean and SD values in the figure legends (indicated with comments boxes “R1 Minor comment 1”).

2. The authors might consider showing movies for some of the data, in addition to the still images.

In our opinion, the still images are sufficient for supporting the paper’s conclusions.

Responses for Reviewer #2:

In Figure 1, the authors looked at stepZ mutants through live imaging of DC and found that the process is significantly slower in the step mutant, which showed some misalignment and holes at the dorsal

midline. *The authors use the appearance of dorsal hairs (DH) as a developmental clock (DH) How good is DH as a marker for staging the embryo? Figure 1A shows what is described as sealing and DH onset, but I can't make out DH in the red square in control embryos. Maybe that is the point, the frame showing the moments before DH? How long does it take for the DH population to emerge completely? Have the authors checked to see if step has any effect on DH?*

In Figure 1A, time zero (or "DH onset") for the control and *step* mutant embryos is designated as the time point immediately before the time point when dorsal hairs became noticeable (the interval between these time points was ~4min). A magnified example of tissue with dorsal hairs is shown for the mutant in the inset at 1:16. The time of dorsal hair onset was closely correlated with sealing onset across control embryos (Fig 1B). The time from dorsal hair onset to half-way dorsal closure was also relatively consistent across control embryos (Fig 1B), and significantly less than the times for *step* mutants (Fig 1B). In *step* mutants, dorsal hairs became noticeable within the same time interval of ~4min, and at halfway closure the dorsal hair signal intensity of the F-actin probe Moe-ABD-GFP was indistinguishable between controls and *step* mutants (Fig 1C).

We included the assessment of dorsal hairs to support two conclusions: (1) the initiation of sealing of the posterior canthus was delayed in *step* mutants versus controls, and (2) the times from sealing initiation to halfway sealing of the dorsal midline were indistinguishable between *step* mutants and controls. Additional information supports these conclusions. A defect in initial sealing of the posterior canthus of *step* mutants was also apparent from an abnormal tissue depression/indentation that arose at the canthus and then resolved once sealing began (Fig 1A and Fig 2A; topic of next reviewer comment). The time period from initial sealing to halfway closure was determined independently of dorsal hairs (Fig 1B).

To clarify the text, revisions were made at the following lines (indicated with comment boxes "R2 comment 1"):

Line 198: "inset shows the time point ~4 min before the detection of dorsal hairs"

Line 203: "Additionally indicating a delay to the initiation of sealing, dorsal hairs formed over the epidermis as the sealing defects persisted."

Line 215: "0:00 (H:MM format) designates the time point before dorsal hair (DH) appearance (insets show the lack of dorsal hairs) and dorsal hairs were detected in the next time point (embryos were imaged every ~4 min)."

The authors describe a group of cells at the posterior end of the amnioserosa in step mutants that lack F-actin and refer to these cells as "depressed". What is meant by this? The lack of F-actin in the "depressed" cells could affect their contractility and could this delay canthus closure? Indeed, these cells are highly constricted as can be seen In Fig 2. There are genes which show elevation in these canthus cells under wild-type conditions and it is worth checking if there is any Step expression in the amnioserosa too. Do any stepMZ embryos make it to DC ?

We describe this *step* mutant abnormality as a "local depression of the epidermal LE and amnioserosa" because in control embryos the apical domains of amnioserosal and epidermal cells exist in a similar plane across the canthus region, whereas a depression/indentation of the embryo surface develops at this site in *step* mutants. The lack of F-actin signal at this site is due to the cells being at lower confocal

imaging planes than the surrounding tissue (a structural defect shown in more detail with the insets of Fig 2A, and indicated with brackets within these insets). We did not explain this aspect of the mutant phenotype with enough detail, and have expanded our explanations in the paper. Revisions were made at the following lines (indicated with comment boxes "R2 comment 2"):

Line 201: "the indentation of the embryo surface was detected as a local absence of signal in the confocal planes shown in Fig 1A, red arrowhead; seen for 7/11 embryos; also see further analysis in Fig 2A"

Line 206: "At the posterior canthus, the abnormal local depression of the embryo surface resolved as sealing initiated (Fig 1A, black arrowhead)."

Line 219: ", and an abnormal depression of the embryo surface (red arrowhead) was at the posterior canthus."

Line 272: "Black box and red arrowhead indicate the abnormal local depression/indentation of the *step* mutant embryo surface at the posterior canthus. Magnified three dimensional analyses of the tissue in the black box (right) show structural details of the abnormal depression (the bracket within the X-Z view indicates amnioserosa tissue bending down to the base of the depression, and the brackets in the X-Y single section and projection provide a reference for detecting a greater area of amnioserosa in the projection which contains additional planes below the X-Y single section)."

We have shown by immunofluorescence (West et al, Current Biology 2017) and imaging of an endogenously expressed GFP fusion protein (Zheng et al, Molecular Biology of the Cell 2019) that Step is expressed in amnioserosa cells.

Step maternal-zygotic mutants display substantial disruptions to early embryo cleavage (Lee and Harris, Current Biology 2013) and these earlier defects might confound analyses of dorsal closure, although we have not yet examined later development of these mutants.

Figure 1 panel E. The dorsal midline in this step mutant does not look like the typical row of cells seen in wild type, instead there are a number of rosettes, which are possibly left over from earlier failed regulation of morphogenesis by Step. Remarkably, the dorsal epidermis has managed to finish the seal despite this, and again this suggests the importance of the amniosera in pulling the dorsal hole shut. The authors went on to directly image the F-actin levels in step mutants versus sibling embryos. The images are not very clear, and this may be one case where the original fluorescent images might be better than the inverted ones. The depressed cells at the posterior canthus are shown again, and again it is not clear what we are looking at. It might be helpful if these cells were looked at with a cortical marker that is itself not affected by dorsal closure, for example anti-phosphotyrosine.

Please note that F-actin levels were specifically affected along the epidermal leading edge, and not other regions of the mutant embryos. Red text and arrows have been added to Fig 2A to more clearly indicate the reduction to F-actin levels at the leading edge and sealing border in the mutants versus controls. Regarding the local depression, please see our response the reviewer's previous comment.

The authors hypothesize that at least some of the DC defects in step mutant embryos are due to myosin mis-regulation. The used ubiquitous expression of constitutively active MLCK to increase myosin activity

and compared the phenotypes generated with those of step mutants but phenotypes differed at the LE. The authors then tried the opposite, reducing the levels of myosin heavy chain with a zip allele and assessing phenotypes with cuticle preps in a step mutant background. The results indicate some rescue of step mutant phenotypes. Interestingly as part of this analysis the authors live imaged step phenotypes with the Moe-ABD-GFP. In Figure 3E it looks like the reporter is coming on in a segmental pattern in the amnioserosa in SteKO mutants; this may be just an artefact of the way the image was cropped or could involve a segmentation gene whose stripes of expression extend around the embryo (eg. paired).

Another explanation for the accumulation of the F-actin probe in some amnioserosa cells and not others are the assembly-disassembly cycles of apical actomyosin networks that occur in these cells (in a single time point, the networks can be present in some cells and absent in neighbouring cells). We have reported that these networks are not obviously affected in *step* mutants (West et al, Current Biology 2017).

Much of the rest of the manuscript is conserved with the genetic analysis of interactions between step and ena, which should provide a nice complement to cell studies. On of the more interesting results is the appearance of LE-like cells behind the LE when Step is overexpressed. It would be interesting to stain with other LE markers to how differentiated these cells are.

We agree that further characterization of the effects of Step over-expression would be interesting. However, the first author of this paper graduated from the lab. Thus, this additional characterization is not feasible for the current paper. Although Step localizes at the ectopic protrusions it induces, we have revised the paper the acknowledge that Step over-expression could have other effects on the cells. Please see line 516 (indicated with comment box "R2 comment 5"):

"However, our data cannot exclude the possibly of Step overexpression inducing ectopic protrusions through separate effects on the cells (e.g. effects on cell differentiation)."
