# Supplementary material for: The Arf-GEF Steppke promotes F-actin accumulation, cell protrusions and tissue sealing during Drosophila dorsal closure
Source: PLoS One. 2020 Nov 13;15(11):e0239357. doi: 10.1371/journal.pone.0239357 (PMC7665897; doi:10.1371/journal.pone.0239357)
Supplement: Attachment — Submitted filename: West-Harris-a.docx [file pone.0239357.s001.docx]

This manuscript comes from a group with an excellent track record in studying regulation of tissue morphogenesis during organismal development. Among the systems they use is dorsal closure of the Drosophila embryo, a nice genetic model for wound healing and developmental epithelial closure. In the present manuscr­­ipt the authors characterized Steppke (step), a cytohesin Arf-GEF that promotes actin polymerization in cell culture, but which in whole animals hinders actomyosin function and the authors wanted to address this further in a whole organism approach.

In Figure 1, the authors looked at *step^Z^* mutants through live imaging of DC and found that the process is significantly slower in the *step* mutant, which showed some misalignment and holes at the dorsal midline. The authors use the appearance of dorsal hairs (DH) as a developmental clock (DH) How good is DH as a marker for staging the embryo? Figure 1A shows what is described as sealing and DH onset, but I can’t make out DH in the red square in control embryos. Maybe that is the point, the frame showing the moments before DH? How long does it take for the DH population to emerge completely? Have the authors checked to see if step has any effect on DH?

The authors describe a group of cells at the posterior end of the amnioserosa in *step* mutants that lack F-actin and refer to these cells as “depressed”. What is meant by this? The lack of F-actin in the “depressed” cells could affect their contractility and could this delay canthus closure? Indeed, these cells are highly constricted as can be seen In Fig 2.­­There are genes which show elevation in these canthus cells under wild-type conditions and it is worth checking if there is any Step expression in the amnioserosa too. Do any step^MZ^ embryos make it to DC ?

Figure 1 panel E. The dorsal midline in this step mutant does not look like the typical row of cells seen in wild type, instead there are a number of rosettes, which are possibly left over from earlier failed regulation of morphogenesis by Step. Remarkably, the dorsal epidermis has managed to finish the seal despite this, and again this suggests the importance of the amniosera in pulling the dorsal hole shut. The authors went on to directly image the F-actin levels in step mutants versus sibling embryos. The images are not very clear, and this may be one case where the original fluorescent images might be better than the inverted ones. The depressed cells at the posterior canthus are shown again, and again it is not clear what we are looking at. It might be helpful if these cells were looked at with a cortical marker that is itself not affected by dorsal closure, for example anti-phosphotyrosine.

The authors hypothesize that at least some of the DC defects in step mutant embryos are due to myosin mis-regulation. The used ubiquitous expression of constitutively active MLCK to increase myosin activity and compared the phenotypes generated with those of step mutants but phenotypes differed at the LE. The authors then tried the opposite, reducing the levels of myosin heavy chain with a zip allele and assessing phenotypes with cuticle preps in a step mutant background. The results indicate some rescue of step mutant phenotypes. Interestingly as part of this analysis the authors live imaged step phenotypes with the Moe-ABD-GFP. In Figure 3E it looks like the reporter is coming on in a segmental pattern in the amnioserosa in Ste^KO^ mutants; this may be just an artefact of the way the image was cropped or could involve a segmentation gene whose stripes of expression extend around the embryo (eg. paired).

Much of the rest of the manuscript is conserved with the genetic analysis of interactions between step and ena, which should provide a nice complement to cell studies. On of the more interesting results is the appearance of LE-like cells behind the LE when Step is overexpressed. It would be interesting to stain with other LE markers to how differentiated these cells are.

The
